# Algebraic Invariants for Inferring 4-Leaf Semi-Directed Phylogenetic Networks

**DOI:** 10.1093/sysbio/syaf071

**Published:** 2025-10-14

**Authors:** Samuel Martin, Niels Holtgrefe, Vincent Moulton, Richard M Leggett

**Affiliations:** European Bioinformatics Institute (EMBL-EBI), Wellcome Genome Campus, Hinxton, Cambridge CB10 1SD, UK; Earlham Institute, Norwich Research Park, Norwich NR4 7UZ, UK; Delft Institute of Applied Mathematics, Delft University of Technology, Mekelweg 4, 2628CD Delft, The Netherlands; School of Computing Sciences, University of East Anglia, Norwich Research Park, Norwich NR4 7TJ, UK; Earlham Institute, Norwich Research Park, Norwich NR4 7UZ, UK

**Keywords:** Phylogenetic invariants, phylogenetic network, semi-directed network

## Abstract

A core goal of phylogenomics is to determine the evolutionary history of a set of species from biological sequence data. Phylogenetic networks are able to describe more complex evolutionary phenomena than phylogenetic trees but are more difficult to accurately reconstruct. Recently, there has been growing interest in developing methods to infer semi-directed phylogenetic networks. As computing such networks can be computationally intensive, one approach to building such networks is to puzzle together smaller networks. Thus, it is essential to have robust methods for inferring semi-directed phylogenetic networks on small numbers of taxa. In this paper, we investigate an algebraic method for performing phylogenetic network inference from nucleotide sequence data on 4-leaf semi-directed phylogenetic networks by analyzing the distribution of leaf-pattern probabilities. On simulated data, we found that we can correctly identify with high accuracy the undirected phylogenetic network for sequences of length at least 10 kbp. We found that identifying the semi-directed network is more challenging and requires sequences of length approaching 10 Mbp. We are also able to use our approach to identify treelike evolution and determine the underlying tree. Finally, we employ our method on a real data set from *Xiphophorus* species and use the results to build a phylogenetic network.

Phylogenetic networks describe the evolutionary history of taxa where reticulate evolution events, such as hybridization and horizontal gene transfer, have occurred (Bapteste et al. 2013). Biologists are becoming increasingly aware that such events are common in the evolutionary histories of many species, and so the development of methods for constructing phylogenetic networks from biological data is an active area of research.

Over the past decades, many methods of phylogenetic network reconstruction have been suggested. One approach is to infer implicit networks that do not aim to represent specific biological processes. For example, distance-based methods such as Neighbor-Net (Bryant and Moulton 2004) construct split networks directly from a distance matrix without the need for sequence data. Other methods construct networks by analyzing gene trees (Than et al. 2008), concordance factors (Allman et al. 2019), or quartets (Grünewald et al. 2013) and attempt to find the “best” network that displays these. Several maximum parsimony algorithms have also been developed for phylogenetic networks (Kanna and Wheeler 2012). An alternative approach is to place an evolutionary model on an explicit network (where each vertex in the network represents a biological event or ancestral species), thereby creating a rooted, directed phylogenetic network. Methods such as maximum likelihood (e.g., Wen et al. 2018; Lutteropp et al. 2022) or Bayesian inference (e.g., Zhang et al. 2018) can then be used to determine how well a set of data fits a certain model and thereby construct a phylogenetic network.

Recently, there has been increasing interest in inferring semi-directed phylogenetic networks for evolutionary analysis (e.g., Solís-Lemus and Ané 2016; Solís-Lemus and Bastide 2017; Allman et al. 2019; Gross et al. 2021; Linz and Wicke 2023). These are networks in which only some of the edges are directed, and these directed edges usually indicate reticulate events (see, e.g., Fig. 1). Semi-directed phylogenetic networks can be inferred from sequencing data by maximizing a likelihood function, but for larger networks, performing a full search of the parameter space of a semi-directed model to determine the parameters that maximize a likelihood function is often too computationally intensive to be practical. One solution to this problem is to use pseudolikelihood, which is based on the likelihood formulas of the 4-taxon subnetworks, as in Solís-Lemus and Ané (2016). Another approach is to build such networks from knowledge of the networks displayed by a small number of taxa. This approach has been used in the past for trees (e.g., Schmidt et al. 2002) as well as explicit networks (Oldman et al. 2016) and more recently for semi-directed networks (Huebler et al. 2019; Allman et al. 2025; Frohn et al. 2025; Holtgrefe et al. 2025). One of the key challenges to this approach is to accurately determine each subnetwork displayed by small numbers of taxa. Here, we attempt to address this challenge.

**Figure 1.**
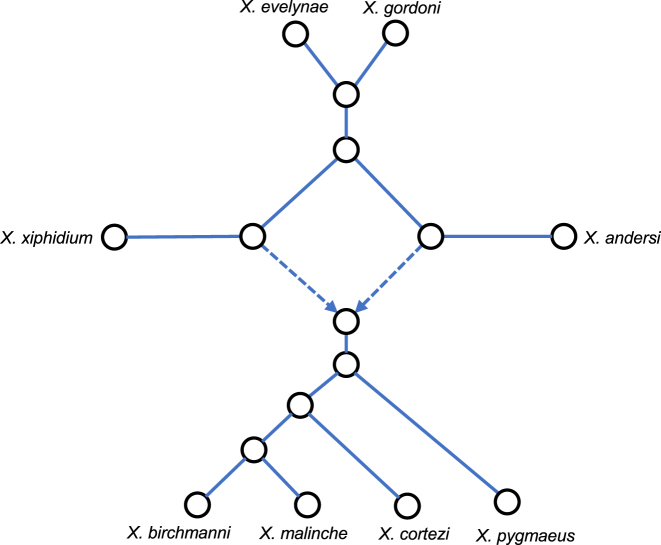
A semi-directed network with eight leaves, labeled by *Xiphophorus* species, constructed from figure 10 of Solís-Lemus and Ané (2016).

For phylogenetic trees, algebraic techniques based on phylogenetic invariants have been used both for the understanding of evolutionary models and for methods of phylogenetic tree inference (e.g., Casanellas and Fernández-Sánchez 2007; Chifman and Kubatko 2014). Recently, algebraic methods have also been used to determine when hybridization between species is likely to have occurred (Blischak et al. 2018) and combined with statistical learning techniques to infer small semi-directed networks (Barton et al. 2022). In this paper, we investigate a method for determining 4-leaf semi-directed networks that uses algebraic invariants for “group-based” models of evolution, namely, the Jukes-Cantor (JC) model and the Kimura 2-parameter (K2P) model. By identifying the state space of the four nucleic acids with a mathematical structure called a group, group-based models enable a Fourier transformation of the parameter space that simplifies the equations defining the model. This, and their low number of parameters, makes them amenable to the algebraic methods we use here. Furthermore, these models are commonly used to model nucleotide substitution in the presence of reticulate evolution (e.g., Burbrink and Gehara 2018; Kong et al. 2024).

As well as performing extensive simulations to understand the performance of the method under various models, we show that it can be used to distinguish between treelike and non-treelike evolution. We also compare our method with the QNR-SVM method (Barton et al. 2022) and employ our method on a real data set that has been previously analyzed using semi-directed networks in Solís-Lemus and Ané (2016) and separately in Blischak et al. (2018) to compare its performance with these methods.

## Materials and Methods

### Background

For a rooted phylogenetic network, its *semi-directed network* is the mixed graph obtained by unrooting the network and undirecting all edges except for the reticulation edges. For the nucleotide substitution models, we define on rooted phylogenetic networks, only the semi-directed network is identifiable from the leaf-pattern distribution (also called the marginal character distribution), that is, the distribution of nucleotides observed at the leaves (Gross et al. 2021). This is analogous for the case for phylogenetic trees, where, under the nucleotide substitution models we use, only the unrooted tree topology is identifiable from the leaf-pattern distribution. Here, we only consider *level-1* phylogenetic networks. These are phylogenetic networks in which the reticulation vertices are sufficiently far away from each other. More precisely, they are phylogenetic networks in which the undirected cycles do not overlap.

We place a model of nucleotide substitution on a rooted phylogenetic network in the form of a directed graphical model, by assigning a transition matrix to each edge in the network (where each entry in the matrix is the probability of a particular nucleotide substitution occurring along that edge), a distribution of nucleotides at the root of the network (for us this will be the equilibrium distribution of the model), and for each reticulation vertex, the probability that a particular position is inherited along either reticulation edge. We refer to this final parameter as the “tree-ratio,” because when it is either 0 or 1, the model becomes that of an unrooted tree. From this model we can obtain expressions for the probability of observing the leaf-patterns (or marginal characters) of the network. Each leaf-pattern is a sequence of nucleotides that can be observed at the leaves of the network at a single position in a sequence alignment.

By considering the numerical parameters of the model as free variables, we think of the distribution of leaf-patterns as a multidimensional polynomial function. These functions are complicated, but they can be simplified if we use certain evolutionary models called “group-based” models (see, e.g., chapter 15 of Sullivant 2018 for further details). For 4-state nucleotide models, there are three well-known group-based models: the JC model, the K2P model, and the Kimura 3-parameter (K3P) model. In each case, a Fourier transformation can be applied to the model that makes the transformed expressions for the distribution of marginal characters much simpler, although it is no longer probabilistic. These transformations were first described for phylogenetic trees in Evans and Speed (1993) and Hendy and Penny (1996), where the transformed distribution functions are monomial (a polynomial with a single term), which makes them especially amenable for algebraic study, and indeed, these models have been well studied from an algebraic perspective (e.g., Sturmfels and Sullivant 2005; Allman et al. 2011).

From an algebraic perspective, we view the phylogenetic tree or network and substitution model as an algebraic variety (see, e.g., Cox et al. 2007 for an introduction). This object can be thought of as a high-dimensional surface, and consists of all possible distributions of leaf-patterns that can be observed from the model. Recent study of these objects has given identifiability results. For the JC model, it was shown that the semi-directed network topology of large-cycle networks is generically identifiable from the distribution of leaf-patterns (Gross and Long 2018). Analogous results have been proven for the K2P and K3P evolutionary models (Hollering and Sullivant 2021), and for all three evolutionary models on level-1, triangle-free phylogenetic networks (Gross et al. 2021). Further algebraic properties have been determined for any triangle-free level-1 network under any group-based model (Gross et al. 2024). In particular, for group-based evolutionary models, it is not possible to identify the reticulation vertex in a 3-cycle from the leaf-pattern distribution (Gross et al. 2021). More recently, for the JC model the triangle-free property on level-1 networks has been relaxed to enable identifiability of the number of reticulation vertices in a level-1 network, up to placement of reticulation vertices on triangles, and identifiability results on some classes of level-2 phylogenetic networks have been obtained (Englander et al. 2025).

Algebraic invariants (also called phylogenetic invariants) are polynomial functions that evaluate to 0 on all points of an algebraic variety given by a fixed phylogenetic network and model of evolution. (Note that the term “phylogenetic invariants” is sometimes used to mean only those algebraic invariants that belong to exactly one tree or network, as in Casanellas and Fernández-Sánchez 2007.) They can be used to determine whether a set of data could have been produced by a given network without the need for parameter estimation. One of the most well-known examples of algebraic invariants are the edge invariants (Allman and Rhodes 2007). These encode the set of splits in a phylogenetic tree from which the full tree can be reconstructed and give rank conditions on certain matrices called flattening matrices. In some circumstances, rank conditions on flattening matrices have been shown to also hold for phylogenetic networks (Casanellas and Fernández-Sánchez 2021). The use of rank conditions on flattening matrices to reconstruct phylogenetic trees from sequence data was employed in the software SVDQuartets (Chifman and Kubatko 2014).

Algebraic invariants have also been used to infer 4-leaf trees from simulated data under the K3P model (Casanellas and Fernández-Sánchez 2007) and 4-leaf networks under the JC model (Barton et al. 2022). Used as a method of inferring tree or network topologies from aligned sequence data, they have several advantages. First, finding the invariants for a fixed phylogenetic tree or phylogenetic network and model of evolution need only be done once. For small trees, many invariants have already been calculated and are available online (Casanellas et al. 2005). Second, using invariants is a statistically consistent method to infer an unrooted phylogenetic tree or semi-directed network topology, provided that an appropriate set of invariants are used. Third, once the invariants have been calculated, applying the method to a data set is simply a case of evaluating a fixed number of polynomials, and so can be performed quicker than many other statistically consistent methods, such as maximum likelihood.

**Figure 2.**
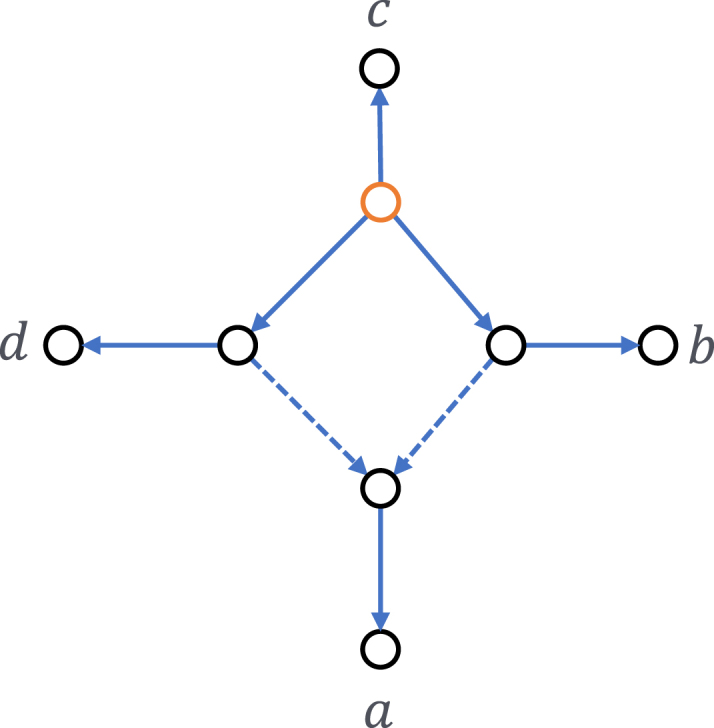
A directed cycle network with cycle of length 4. Dashed edges represent reticulation edges. All edges are directed away from the root vertex, which sits below leaf *c*. This topology could represent the evolutionary history between taxa, where *a* is a hybrid species resulting from a hybridization event between taxa *b* and *d*, while *c* is an outgroup.

Here, we investigate the *practical identifiability* of semi-directed networks and the effectiveness of using algebraic invariants for network inference under the JC and K2P nucleotide substitution models. We present a new algorithm to infer the 4-leaf 4-cycle network (also called a 4-*sunlet* network, a directed example of which is depicted in Fig. 2) from aligned sequence data using algebraic invariants. The algorithm is based on that developed for 4-leaf trees in Casanellas and Fernández-Sánchez (2007). The 4-leaf 4-cycle network has particular biological relevance in that it can represent the evolutionary relationship between two species, their hybrid, and an outgroup; and it is generically identifiable from leaf-pattern data. Furthermore, knowledge of all the networks restricted to 4-taxa subsets, called “quarnets,” is sufficient to rebuild a (level-1) phylogenetic network. When the network is assumed to be triangle-free, we need only 4-cycles and 4-leaf trees to rebuild the network (Frohn et al. 2025; Huber et al. 2025).

### Algorithm to Infer Phylogenetic Network Topology from Aligned Sequence Data

We developed an algorithm that utilizes phylogenetic invariants to infer the correct 4-leaf 4-cycle network from a multiple sequence alignment (MSA) of four taxa. We use the notation $( {\textit{abcd}} )$ to denote the 4-leaf 4-cycle network with taxon “*a*” at the leaf below the reticulation vertex and taxa “*b*”, “*c*,” and “*d*” at the leaves going anti-clockwise from “*a*” (as in Fig. 2). There are $12 = 4!/2$ possible 4-leaf 4-cycle networks, because $( {\textit{abcd}} )$ and $( {\textit{adcb}} )$ represent the same 4-cycle. Note that for each network the underlying semi-directed graph is the same, but the taxa labels at the leaves are permuted.

**Figure 3.**
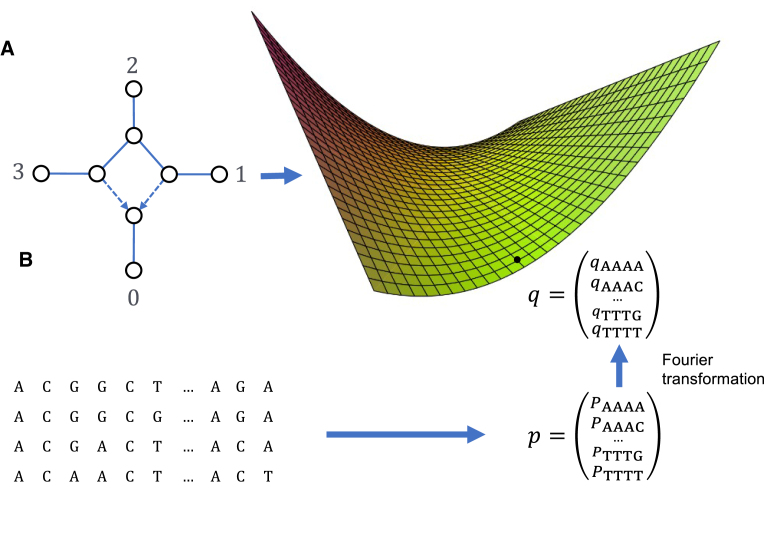
(A) Each leaf-labeled, semi-directed network is converted into a high-dimensional “surface” in a high-dimensional space (depicted here as a 2D surface in a 3D space), representing all leaf-pattern distributions possible from the model. (B) An MSA is converted into a point *q* in this space, and this lies exactly on the surface (i.e., the alignment could have been generated by the model) if and only if $f( q ) = 0$ for all invariants *f*. Because each *f* is a continuous polynomial, for points *q* close to the surface, $f( q )$ will be close to 0. Depiction of the surface was created using CalcPlot3D available at https://c3d.libretexts.org/CalcPlot3D/index.html.

Each of the 4-leaf 4-cycle networks is represented by a “surface” giving the leaf-pattern distributions that can be obtained from that network and substitution model. From an MSA from four taxa we obtain an empirical leaf-pattern distribution, and we use algebraic invariants to determine how close this is to each of the surfaces. A depiction of this process is given in Figure 3.

First, using the software Macaulay2 (Grayson and Stillman 2002), we calculated invariants for the 4-leaf 4-cycle phylogenetic network depicted in Figure 2, for the JC and K2P models. Full details on how these were calculated and which invariants were chosen are given in the Supplementary Materials. We now briefly describe our algorithm for inferring a phylogenetic network topology from aligned sequence data. Further details and a full description are given in the Supplementary Materials. For a given MSA, we score each of the 12 semi-directed network topologies by permuting the sequences in the MSA, applying the Fourier transform to the corresponding leaf-pattern distribution, and then applying invariants to the result. The first step is to read the alignment and count the number of columns that occur for each leaf-pattern. This gives us an empirical leaf-pattern distribution which we store as a single vector *p*. The next step is to transform *p* using the Fourier transformation, giving us a new vector *q*.

Our algorithm next reads in a file of invariants that have undergone the Fourier transformation as above, and we evaluate each invariant at the vector *q*. This gives us a list of numbers from which a score for the corresponding network is given. As in Casanellas and Fernández-Sánchez (2007), we found that scoring using the 1-Norm gave us the best results. In this case, the score for network $\mathcal{N}$ is given by the formula


\begin{eqnarray*}
{S_\mathcal{N}} = \,\,\mathop \sum \limits_{f \in G} \left| {f\left( q \right)} \right|,
\end{eqnarray*}

where *G* is a set of invariants and *q* is the transformed data point obtained from the permuted MSA. Once each network has been scored, the networks are ordered by score in ascending order, and the network with the smallest score is chosen as the one most likely to have generated the data.

It is common to infer phylogenetic trees or networks from a single MSA. In this case, it is desirable to have an idea of the confidence a tool has in its inference. Bootstrap support (Felsenstein 1985) is a popular method of providing confidence intervals for phylogenetic inferences. Here, we sample with replacement many times from the original data to create new data sets with similar properties. In our case, we expect the new data sets will have leaf-pattern distributions close to that of the original data set. We implemented a separate version of our script with inbuilt support for parallelized bootstrapping. We applied our bootstrap method to real transcriptome data from 24 swordtail fish and platyfish species (genus *Xiphophorus*) and two outgroups (*Pseudoxiphophorus jonesii* and *Priapella compressa*), by independently assessing each subset of four taxa. The transcriptome data were generated in Cui et al. (2013) and alignments were kindly provided to us by the authors of Blischak et al. (2018).

We implemented our inference algorithm in a python script evaluate.py (and with bootstrap in evaluate_bootstrap.py) along with a python library for reading, writing, and evaluating phylogenetic invariants. These are available from the GitHub repository https://github.com/SR-Martin/4cycle_invariants.

## Results

In this section, we evaluate our method on simulated data, compare its performance to QNR-SVM, and demonstrate its utility on real data. All data were simulated using the simulation scripts available on the GitHub page. Unless otherwise stated, the results in this section are obtained using invariants from the appropriate model, as described in the Supplementary Materials.

### Simulated 4-Leaf 4-Cycle Data

**Figure 4.**
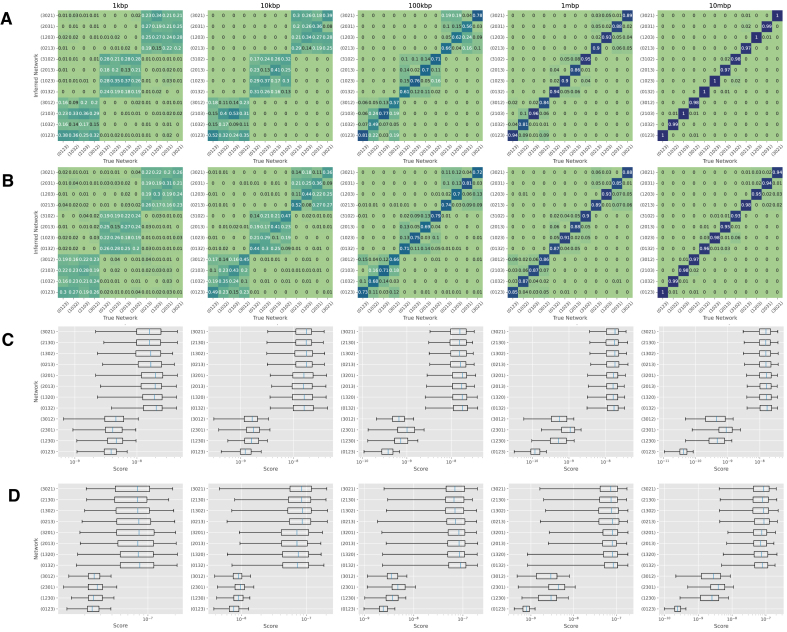
(A) Confusion matrices for inference of 4-leaf 4-cycle networks from data simulated under the JC model. (B) Confusion matrices for inference of 4-leaf 4-cycle networks from data simulated under the K2P model. (C) Distribution of scores from data generated by the network (0123) under the JC model. (D). Distribution of scores from data generated by the network (0123) under the K2P model

We generated MSA data from each of the 12 distinct leaf permutations of the directed network depicted in Figure 2, each of which has a semi-directed network identifiable from leaf-pattern data. For each network, we generated 100 MSAs of lengths 1 kbp, 10 kbp, 100 kbp, 1 Mbp, and 10 Mbp under both the JC and K2P models. For each edge, substitution rates were generated uniformly at random in the interval (0, 0.1) for JC and (0, 0.15) for K2P. The tree ratio ($\gamma $) was fixed at 0.5. Each MSA was assessed using the algorithm described in the *Algorithm to Infer Phylogenetic Network Topology from Aligned Sequence Data* section, and in each case the 4-cycle topology with the lowest score was taken as the “inferred network.” Figure 4A shows the confusion matrices for these data sets for the JC model, and Figure 4B shows the confusion matrices for the K2P model. In both cases, we see that we approach 100% true positive and 0% false positive rates as the sequence length approaches 10 Mbp. Furthermore, we can see the set of 4-leaf 4-cycle networks is partitioned into three sets, where in each set the circular order is the same (e.g., the first set is given by (0123), (1230), (2301), and (3012)). Figure 4A and B shows that at lengths of 1kbp, we can identify the correct circular order with close to 100% true positive rate. In this case, identifying the circular order is equivalent to identifying the *undirected phylogenetic network*.

We found a clear distinction between scores for the true network and scores for the other networks. Figure 4C and D shows the distribution of scores obtained for each network when the true network was $( {0123} )$, under the JC and K2P models, respectively. Again, we can see that scores for networks with the correct circular order are smaller than scores for all others from 1 kbp. At each alignment length, we see that the mean score of the true network is smaller than the mean scores of all others, suggesting a bootstrap approach might be beneficial (see the *Inference of Networks from Real Data Sets with Many Taxa* section). At 1 Mbp, there is no overlap between the interquartile range of scores for the true network and the interquartile range of scores for all other networks. By 10 Mbp, this effect is more pronounced, and the score for the true network is smaller than the scores for other networks with the same circular order.

### Varying the Tree Ratio

**Figure 5.**
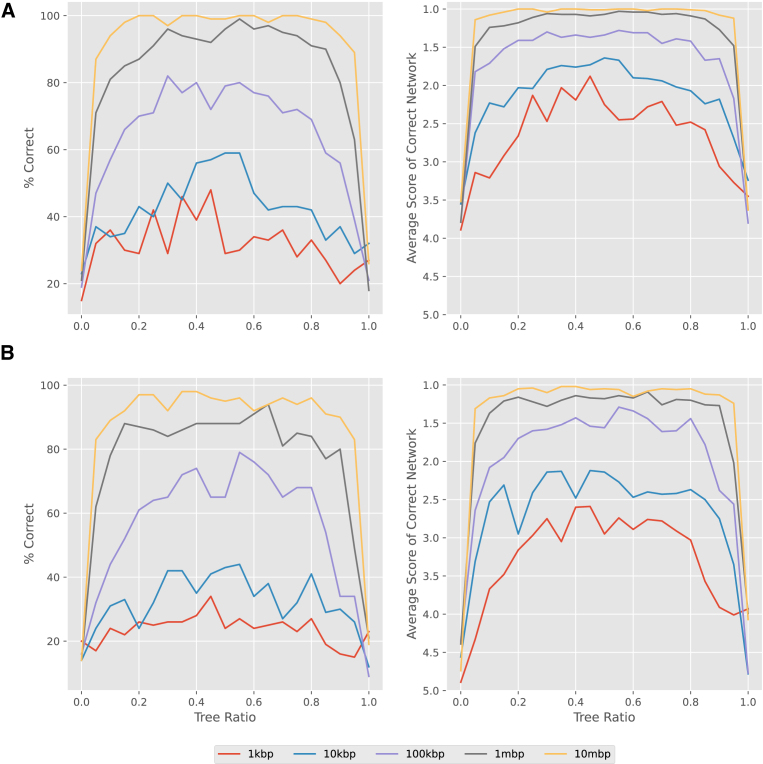
(A) Percent of tree-ratio data sets where the network was correctly identified (left) and average score over all data sets that the correct network achieved (right) for JC. (B) Analogous plots for K2P data sets.

We generated further data sets where the 4-leaf 4-cycle network was fixed and the tree ratio $\gamma $ was varied from 0.0 to 1.0 in intervals of 0.05. As before, we generated 100 MSAs of length 1 kbp, 10 kbp, 100 kbp, 1 Mbp, and 10 Mbp for both JC and K2P models. Figure 5 shows the number of correctly inferred networks in each case for the JC model (5A) and the K2P model (5B). Observe that when $\gamma = 0$ or 1, we obtain a low percent of correctly identified networks regardless of MSA length, because in this case the data are from a phylogenetic tree, and there is not a unique semi-directed phylogenetic network that could have produced it.

### Identification of Treelike versus Non-Treelike Evolution

In many cases, it may not be known whether a set of taxa has undergone reticulate evolution or not. In this section, we focus on using our method to determine whether data from four taxa have been generated from a tree (treelike) or a 4-leaf 4-cycle network (non-treelike).

To do this, we make the following observation: for a fixed model of evolution, the model for a 4-leaf unrooted tree is contained in the models of the eight 4-leaf 4-cycle networks that display the tree, and is not contained in the remaining four. A diagram showing these containments for the 4-leaf tree $( {( {1,2} ),( {3,4} )} )$ can be found in Supplementary Figure S5. Therefore, if a set of data is generated by a tree, we expect the score that this data obtains via our invariants method to be low for eight of the networks, and high for the remaining four networks. Furthermore, from the partition of the networks by their scores, we can determine which unrooted tree generated the data. The scripts evaluate.py and evaluate_bootstrap.py automatically search for this signature and inform the user when it has been found.

**Figure 6.**
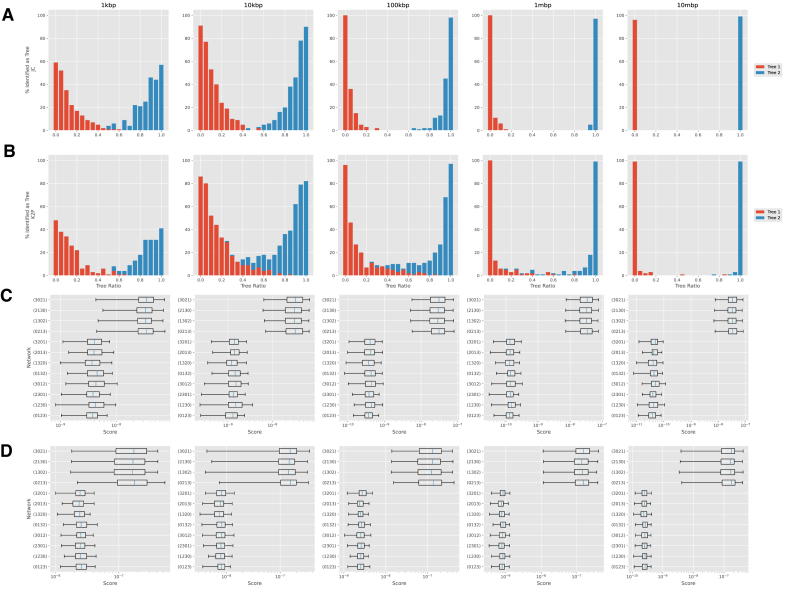
(A) Percent of JC data sets identified as evolving along either tree 1 or tree 2. (B) Percent of K2P data sets identified as evolving along either tree 1 or tree 2. (C) Scores assigned to networks generated along tree 1 (i.e., $\gamma = 0$) under the JC model. The low scores of eight of the networks are clearly identifiable. (D) Scores assigned to networks generated along tree 1 under the K2P model.

We found that this signature is identifiable even for short alignment lengths (see Fig. 6C). In this case, when $\gamma = 0$, evolution has been treelike, along a tree which we refer to as tree 1. When $\gamma = 1$, evolution has also been treelike, along a different tree which we refer to as tree 2. In all other cases, evolution has been non-treelike. Figure 6A shows that for the data simulated under the JC model, we approach a 100% true positive rate and a 0% false positive rate for alignments of length 10 Mbp. Figure 6B shows a similar picture for the data simulated under the K2P model.

### Assessment against QNR-SVM

**Figure 7.**
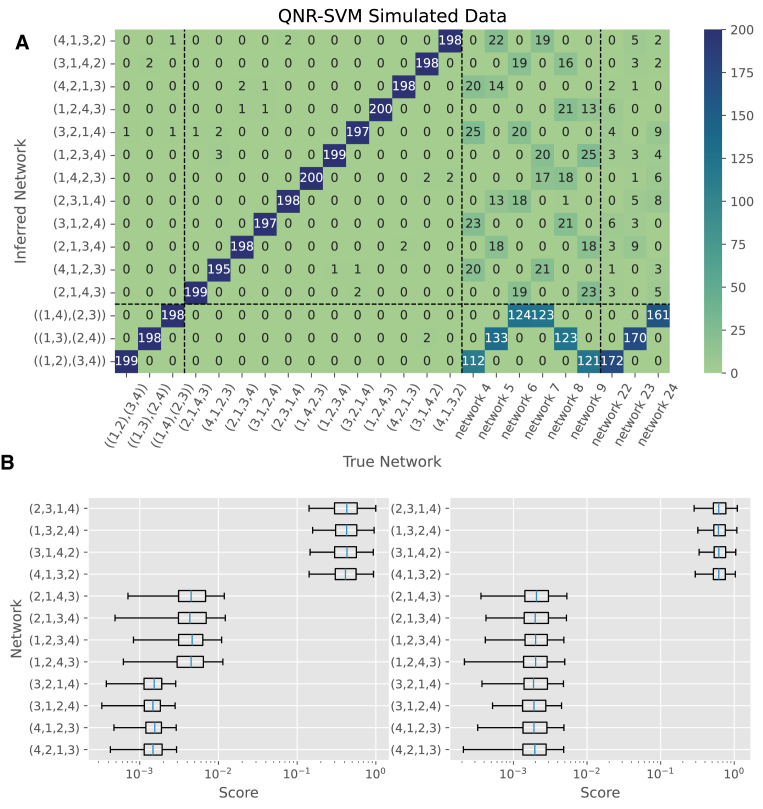
(A) Confusion matrix for analysis of data sets from Barton et al. (2022). The first three columns are data sets simulated from phylogenetic trees. The next 12 columns are from 4-leaf 4-cycle networks. The next six columns, labeled networks 4–9, are 3-cycle networks. The final three columns are double 3-cycle networks (see fig. 5 of Barton et al. 2022). Each column represents 200 data sets. (B) Scores for each 4-cycle network on data from (left) a single 3-cycle (network 4) and (right) a double 3-cycle (network 22).

We assessed our method against the QNR-SVM method presented in Barton et al. (2022). This method uses a support vector machine to analyze the residuals from a set of phylogenetic invariants, one for each identifiable semi-directed phylogenetic network topology on four leaves. In particular, the method can identify undirected 3-cycles (i.e., quarnets that contain 3-cycles in which the reticulation vertex is not identified). We assessed our method on the simulated data available in Barton et al. (2022), from the three unrooted 4-leaf tree topologies (networks 1, 2, and 3 in Barton et al. 2022), the twelve 4-cycle topologies (networks 10–22), the six topologies with a single 3-cycle (networks 4–9), and the three topologies with two 3-cycles (networks 22–24). The data were simulated under a JC model with branch lengths of cycle and cycle-adjacent edges chosen uniformly at random between 0.05 and 0.2, and branch lengths of all other edges chosen uniformly at random between 0.05 and 0.4. The tree-ratio parameter $\gamma $ was chosen uniformly at random between 0.25 and 0.75, and the alignment length was 1 Mbp. The results of our method on this data are displayed in Figure 7.

We found that on trees and 4-cycles, our method gives very similar results to those in Barton et al. (2022) (see fig. 5 therein), with all topologies being identified at close to 100% true positive rate. In particular, here we are able to correctly identify a higher proportion of the 4-leaf trees (lower left box in Fig. 7) than in Barton et al. (2022). However, we note that here we do not attempt to identify phylogenetic networks containing 3-cycles, and these accounted for many of the false positives for the 4-leaf tree data in Barton et al. (2022). We observe that the convergence of our method on this data is an order of magnitude better than the simulated data in the *Simulated 4-Leaf 4-Cycle Data* section.

We do not attempt to identify topologies with 3-cycles, because the placement of the reticulation vertex is not identifiable. However, it is helpful to know how our method performs in these cases. In most cases we infer the 4-leaf tree obtained by collapsing the 3-cycle to a single point (Fig. 7A). Inspection of the scores reveals a similar situation to the scores on tree topologies in the *Identification of Treelike versus Non-Treelike Evolution* section, where particular topologies consistently score lower than others (Fig. 7B).

### Data Simulated under the General Markov Model

Although computationally tractable, the JC and K2P evolutionary models are restrictive in terms of the substitution rates they allow, and may not accurately reflect real-world processes. We assessed our method on data simulated under the general Markov model, which places no restrictions on the form of the transition matrices placed along edges in our network. This model is similar to the unrestricted (UNREST) model (Yang 1994), but here we allow independent rate matrices associated to each edge. As before, we simulated 100 MSAs of length 1 kbp, 10 kbp, 100 kbp, 1 mbp, and 10 mbp, from the 4-leaf 4-cycle network (0123) (as in Fig. 2). For each MSA, transition matrices and the root distribution were randomly generated. To maintain biological plausibility, each transition matrix had substitution rates generated independently at random from a uniform distribution between 0% and 5% (with diagonal entries ensuring the row sum equals 1). The first three entries of the root distribution were generated independently at random from a uniform distribution between 20% and 30%, with the final entry ensuring they summed to 1. In all cases we set the tree ratio $\gamma = 0.5.$

**Figure 8.**
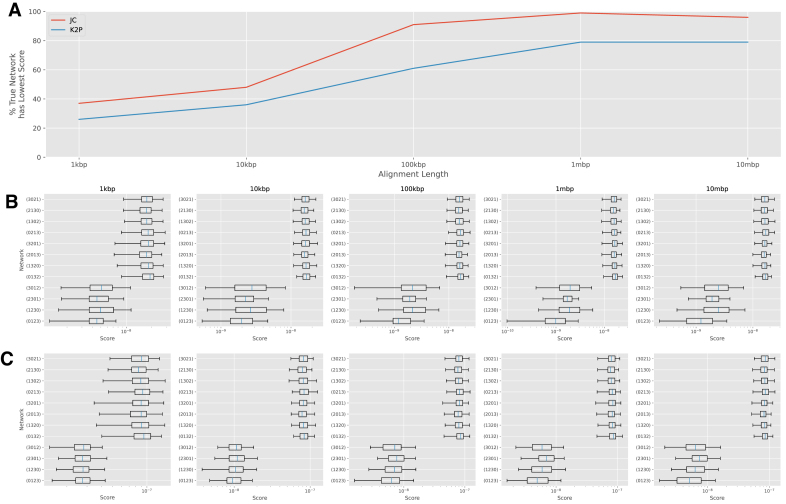
(A) Percent of data sets for which the true network has the lowest score when assessed with the JC and K2P invariants. JC invariants appear to perform better than K2P invariants. (B) Box plots of the scores for each network for JC (first row) and K2P (second row). Scores for the networks with the correct circular order are noticeably lower than others, particularly for the K2P invariants.

We assessed each data set using both the JC and K2P invariants from the previous sections. The results are displayed in Figure 8. In both cases, the true network becomes the lowest scoring network as the MSAs get longer, up to a rate of 99% for the MSAs of length 1 mbp when using the JC invariants (Fig. 8A). As with our previous simulations, we find that networks with the correct circular order score lower than others (Fig. 8B). For both sets of invariants, the scores for these networks show good separation from the scores for other networks, particularly for the K2P invariants. However, unlike in our previous simulations, the score of the true network is not substantially less than others with the same circular order, making it more difficult to place the reticulation vertex correctly.

### Data Simulated under the Network Multispecies Coalescent Model

For a group of species whose evolution is described by a phylogenetic network, gene tree discordance can be caused by genes that evolve along different trees displayed by the species network. However, gene tree discordance can also be caused by phenomena such as incomplete lineage sorting. This is modeled by the multispecies coalescent (MSC) model, which, given a species tree, describes a distribution of gene trees that could be produced by that species tree (Rannala et al. 2020). The network multispecies coalescent model (NMSC) extends the MSC model to allow for species networks that describe events such as hybridization (Degnan 2018). This model is also known as the multispecies coalescent with introgression (MSci) model (Jiao et al. 2021).

In our approach, we model evolution at a single molecular site on a level-1 phylogenetic network, and assume all sites are independent and identically distributed. We therefore do not model the effects of incomplete lineage sorting. Nonetheless, we find that our method has some robustness to data simulated under the NMSC model, and is still able to predict the correct undirected network in many cases.

**Figure 9.**
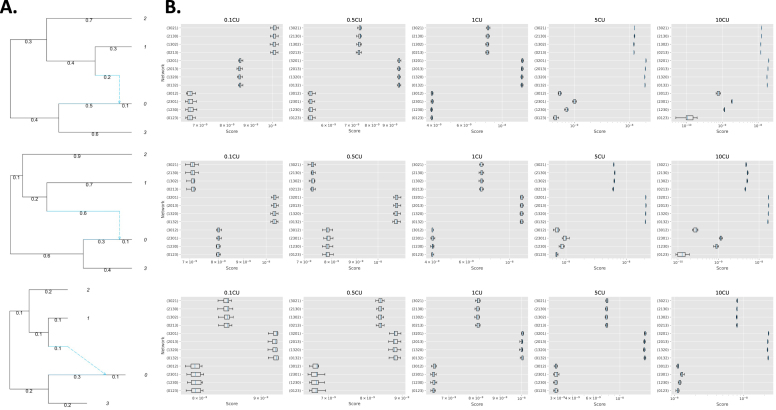
(A) Three different rooted network topologies, produced using PhyloPlot (Ané 2022). (B) Histograms of the scores from our method. Each column corresponds to the scores obtained on the network in that row, with edge lengths multiplied by 0.1, 0.5, 1, 5, and 10 coalescent units (CU) respectively. Each plot shows box plots of the score for each of the 12 semi-directed networks over 10 simulated alignments of length 1 mbp.

We simulated 1000 gene trees under the NMSC model for each of the three directed networks in Figure 9A, using PhyloCoalSimulations (Fogg et al. 2023). Each network represents the history of four species for which a single hybridization event occurred, and in each case the corresponding semi-directed network is the 4-leaf 4-cycle network (0123). The first two networks are ultrametric (all paths from the root to each leaf have equal length). For each network we performed five sets of simulations, by scaling all edge lengths in the network by 0.1, 0.5, 1, 5, and 10. Here, edge lengths are in coalescent units, with shorter edges resulting in a larger gene tree discordance effect coming from incomplete lineage sorting, and longer edges resulting in a larger gene tree discordance effect coming from the hybridization event.

Next, for each set of gene trees, we simulated 10 independent MSAs under the JC model using AliSim (Ly-Trong et al. 2022). Here, each gene had length 1000 bp and the sequences from all 1000 genes were concatenated, for a total length of 1 mbp. To convert from coalescent units to expected number of mutations, we assumed an effective population size ${N_e} = {10^6}$, and a mutation per generation rate $\mu = {10^{ - 8}}$. The exact commands we used for simulation can be found in the Supplementary Materials.

Each MSA was then assessed using our method. We found that when the length from root to tip was at least 1 coalescent unit, we could reliably determine the undirected network, and at 10 coalescent units we could determine the semi-directed network (Figure 9B). As branch lengths got shorter, this was less certain; for networks 1 and 3 we still obtained the correct undirected network, but for network 2 we did not (Figure 9B).

### Inference of Networks from Real Data Sets with Many Taxa

Here, we demonstrate the utility of our method on aligned transcriptome data from 24 swordtail fish and platyfish species (genus *Xiphophorus*) and two outgroups (*Pseudoxiphophorus jonesii* and *Priapella compressa*), generated in Cui et al. (2013). Each of the *Xiphophorus* species belongs to one of three distinct clades: southern swordtails, northern swordtails, and platyfishes (split further into southern platyfishes and northern platyfishes). Because our method is restricted to four taxa, we looked at each subset of four taxa individually, giving a total of 14,950 subsets. The data consist of 10,999 alignments, each of length at least 500 bp, for a total alignment length of 16.85 Mbp. Because we do not use positional information, we concatenated all alignments into a single alignment. Next, we extracted the concatenated alignment for each subset of four taxa. Each of these subsequent alignments was analyzed by our bootstrap method, with 100 bootstrap replicates in each case, using the K2P invariants (see below for rationale). Here, we ignore columns in the alignment containing the gap character “-”. Without gaps, alignments between subsets of four taxa ranged between 180 kbp and 3.37 Mbp.

We then used the software Squirrel (Holtgrefe et al. 2025) to create a level-1 phylogenetic network displaying the relationships between all 24 *Xiphophorus* species. Squirrel is a new approach that can take as input the quarnets computed using the method presented here to build larger level-1 (triangle-free) phylogenetic networks on many taxa. When constructing cycles of length greater than 4 in this network, Squirrel uses only the circular ordering of the 4-sunlets, and not the position of the reticulation. In the previous section, we found that the K2P invariants were most likely to determine the correct circular ordering on data generated under the general Markov model (Fig. 8B), so we chose to use these invariants.

Of the 14,950 4-taxa subsets, 7028 (47%) had 100% bootstrap support for a particular tree (6175) or 4-cycle (853) topology, and 8982 (60.1%) had at least 90% support for a particular tree (6325) or 4-cycle (2,657) topology. Almost all (14,561) had at least 50% support for a particular tree (6459) or 4-cycle (8102) topology, consistent with widespread hybridization between *Xiphophorus* species, as demonstrated in previous analyses of this data set (Cui et al. 2013; Solís-Lemus and Ané 2016; Blischak et al. 2018). The bootstrap results show that the inference of 4-cycles is less certain than the inference of trees, likely due to the difficulty in placing the reticulation vertex, which we observed in the *Simulated 4-Leaf 4-Cycle Data* section. The full results for all 4-subsets are available in the Supplementary Materials.

**Figure 10.**
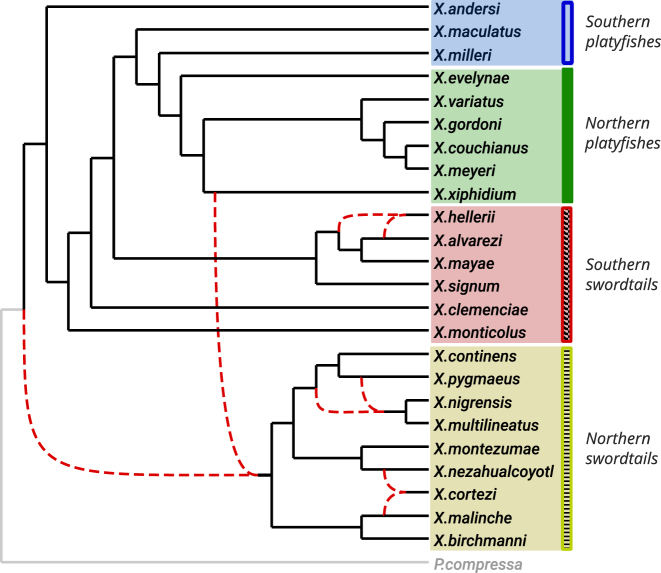
The level-1 phylogenetic network produced by Squirrel, using the bootstrap-supported quarnets from *Xiphophorus* data. Dashed lines indicate reticulation edges.

Next, we created a level-1 phylogenetic network using an adapted version of the software Squirrel. For input, we gave Squirrel the highest-supported tree or 4-cycle network from each 4-subset, and these were weighted by the corresponding support value. Squirrel allows exactly one taxon to be designated the outgroup in order to root the network. We designated *P. compressa* as the outgroup, and therefore excluded all subsets containing *Ps. jonesii*. The network produced by Squirrel is displayed in Figure 10. It shows clear separation between the clades (although the southern swordtails do not form a monophyletic group) and is in agreement with that produced in Solís-Lemus and Ané (2016, see fig. 10 therein). In particular, we find a reticulation event between *Xiphophorus xiphidium* and the northern swordtail clade, exactly as described in Solís-Lemus and Ané (2016) and also reported in Blischak et al. (2018). We also find further reticulation within the northern swordtail clade, in line with the results of Cui et al. (2013). For example, they constructed two trees that placed *Xiphophorus nezahualcoyotl* as sister to *Xiphophorus cortezi* and *Xiphophorus montezumae*, respectively. Here, we find a reticulation event enabling both placements.

### Timings

**Figure 11.**
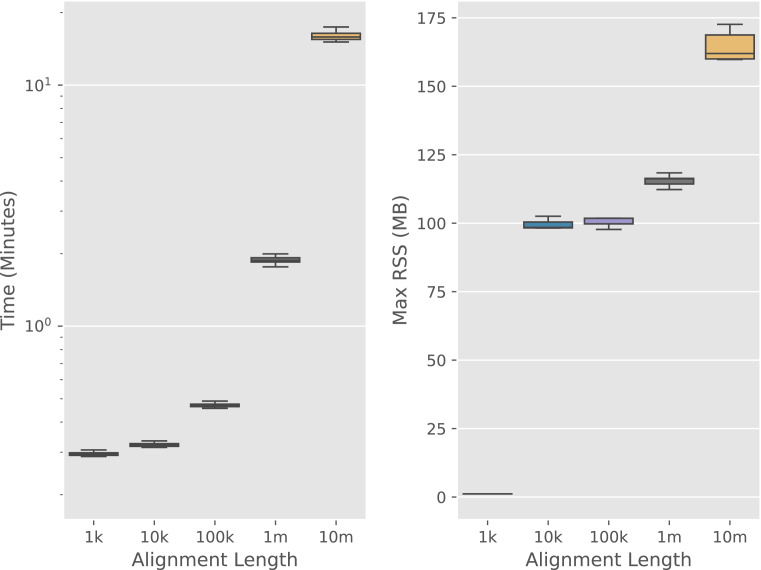
Timings and max memory usage for simulated JC data (*Simulated 4-Leaf 4-Cycle Data* section) split by alignment length.

Figure 11 shows the time taken and maximum memory usage for the analysis of the simulated data from the *Simulated 4-Leaf 4-Cycle Data* section. Each analysis was run on a single CPU with 8 GB of RAM. Because each data set is evaluated on a fixed set of invariants (that have been precomputed and are stored in a text file), most of the time is taken on reading the alignments and counting leaf-patterns to obtain the empirical leaf-pattern distribution, and then performing a Fourier transform of this data. The time therefore scales with the length of the alignment. Shorter alignments perform quickly (seconds), but longer alignments can take several minutes. Memory usage scales with alignment length, as the whole alignment is loaded into memory to calculate the empirical distribution of leaf-patterns. However, this is not necessary and could be improved so that memory usage was fixed by reading alignments piecewise.

## Discussion

We have developed a novel method for inferring a semi-directed network topology from aligned sequence data between four taxa. We demonstrate its use in identifying 4-leaf 4-cycle networks from simulated data, and in identifying whether reticulate evolution is likely to have occurred. We have shown that we can identify the undirected network with sequences of length 1 kbp but require longer sequences (up to 10 Mbp) to determine which vertex is the reticulation vertex and thereby identify the semi-directed network. Furthermore, we show that our method can detect when evolution between taxa has been treelike, converging quickly to a high true positive rate and a low false negative rate as alignment length increases.

On simulated data, we observe a rate of convergence that is much less than the analogous rate for trees. In Casanellas and Fernández-Sánchez (2007), the authors observe almost 100% accuracy for alignments of length 10 kbp on 4-leaf trees under the K3P model. For 4-leaf 4-cycle networks under the JC and K2P models, we do not achieve 100% accuracy until alignment lengths are in the order of 10 Mbp. However, for alignments of length at least 1 kbp, we were able to infer with high accuracy the correct circular ordering of 4-leaf 4-cycle networks and thereby determine the undirected network. There are three circular orderings possible, each corresponding to a choice of two out of three 4-leaf unrooted trees displayed by the network. Thus, when restricting to undirected networks, our results are comparable with those in Casanellas and Fernández-Sánchez (2007). Locating the correct reticulation vertex appears to be the main difficulty. We conjecture that the leaf-pattern distribution varieties of 4-leaf 4-cycle networks with the same circular ordering are close together geometrically. The scores displayed in Figure 4C and D support this, and this makes inference difficult. The varieties corresponding to any two 4-leaf-4-cycle networks contain exactly one variety corresponding to a phylogenetic tree in their intersection, and so for data where the tree ratio is close to 0 or 1, the true semi-directed network topology will be more difficult to infer. This can be observed in Figure 6.

We compared our method with the QNR-SVM method in Barton et al. (2022) and found the performance of our method comparable with theirs on trees and 4-cycles, with true positive and false positive rates very similar. Unless it is known that a data set is similar to data used in the pretrained QNR-SVM model, to use QNR-SVM one must first train the model on the data. The method we present has the advantage that it does not require training and is therefore much quicker to run. We found that the convergence of our method was an order of magnitude better on the QNR-SVM data than on our own simulated data sets, with a true positive rate of almost 100% being achieved on data from alignments of length 1 Mbp. The main difference in the two data sets is that the QNR-SVM 4-leaf 4-cycle networks are less symmetric, with branch lengths having different ranges depending on the branch. A better understanding of the geometry and how this corresponds to the parameter space may enable faster convergence.

Because we do not attempt to identify 3-cycle topologies, we were unable to identify the correct topology for the data in Barton et al. (2022) generated from 3-cycle topologies. Nonetheless, we analyzed this data using our method and inspected the results. In most cases our method inferred the 4-leaf tree obtained by collapsing the 3-cycle(s) to a single vertex (Fig. 7A). When subsequently building a phylogenetic network using Squirrel, this will not affect the result, because Squirrel will collapse 3-cycles to a single vertex on all input quarnets. We found that for each 3-cycle topology, particular 4-cycles scored consistently lower than others, much like the case for 4-leaf trees in the *Identification of Treelike versus Non-Treelike Evolution* section. For the networks with a single 3-cycle, this is expected, because, under the JC substitution model, each 3-cycle model is contained in exactly four of the 4-cycle models (see fig. 10 of Gross and Long 2018). The distribution of scores in this case lies somewhere between the distribution for 4-cycles (Fig. 4C) and the distribution for trees (Fig. 6C), which agrees with the containment results. Thus, a very careful analysis of the scores here may enable us to determine quarnets containing a single 3-cycle, in a similar way to how we determine treelike evolution. For the topologies with two 3-cycles however, we do not have the same containment of models, so the results here are less clear. The distribution of scores in this case was closer to that of the 4-leaf trees. Further work is needed to determine whether we can identify these topologies from the 4-cycle scores.

We also assessed our method on data simulated under different models. Under the general Markov substitution model, we found that both sets of invariants performed well when inferring only the undirected network, but placement of the reticulation vertex was less reliable, even for longer alignments. Under the NMSC model, we found that our method has some robustness to incomplete lineage sorting when this was not the main source of gene tree discordance, and was able to pick out the correct semi-directed network. In some cases, even when incomplete lineage sorting was the main source of gene tree discordance, our method was able to determine the correct undirected network. However, other methods that model the NMSC directly may be more appropriate when incomplete lineage sorting is known to have occurred. For example, PhyNEST (Kong et al. 2024) estimates phylogenetic networks from site-pattern data under an NMSC model with a JC substitution model.

Our method looked at small networks with four taxa. In principle, one can apply the same method to larger networks with more taxa, but the problem of calculating invariants for larger networks is currently intractable. Alternatively, one could construct a larger network by computing networks on smaller numbers of taxa and puzzling them together to make larger networks (see, e.g., Huber and Moulton 2013; Oldman et al. 2016, where directed networks are constructed from 3-leaf networks). Recently, we made some progress in this direction for semi-directed networks by developing a new approach that can take as input the quarnets computed using the method presented here to build larger level-1 (triangle-free) phylogenetic networks on many taxa, or directly from MSAs using a heuristic based on statistical geometry (Holtgrefe et al. 2025). This approach is implemented in the software Squirrel. Here. we applied our new method, combined with bootstrapping, to aligned transcriptome data from swordfish species and used the results as input for Squirrel to create a level-1 phylogenetic network. This network displayed previously identified hybridization events and was largely consistent with previous analyses. Bootstrapping enabled us to give confidence intervals to the tree or 4-leaf 4-cycle networks we inferred, which we then used as weights for the corresponding network when given to the software Squirrel. However, we are only able to give the single most supported topology for each 4-subset to Squirrel. This means that information on other topologies that might be well-supported is lost. Future work on Squirrel will take alternative topologies into account, and we believe will provide more accurate phylogenetic network reconstruction.

In our simulations, we found that we were able to identify the circular ordering of 4-cycles with high accuracy from smaller alignments, whereas identifying the position of the reticulation vertex required longer alignments. However, for constructing larger level-1 phylogenetic networks, Squirrel only uses the placement of the reticulation vertex in a 4-cycle quarnet to place the reticulation vertex in 4-cycles in the final network. For larger cycles in the final network, the circular orderings of the 4-cycle quarnets are used. Thus, we may still be able to create accurate level-1 phylogenetic networks even if we are not able to always identify the correct reticulation vertex, as is the case for shorter alignments.

We developed several python scripts for both simulating and assessing aligned sequence data. These scripts read in plain-text files containing expressions for the phylogenetic invariants to use and may therefore be useful for other researchers assessing other sets of invariants. Our tool performs quickly on all data sets, with time demand growing with alignment length. The computations that take the most time are calculating the empirical distribution of leaf-patterns, followed by performing a linear transformation of this distribution. Both tasks are parallelizable and implementing this could increase the speed by up to 12×, although we have not explored this yet. The remaining time is spent evaluating the polynomial invariants on the transformed frequency data, and this is also parallelizable. Thus, there is potential for our tool to be significantly faster. The reason we can perform network inference relatively quickly is that the most difficult computations (computing the invariants of the networks) need only be done once. We have already done them and distribute the results with the tool. The speed at which this tool runs means that it may be useful for exploratory or initial analyses of large data sets. Indeed, our tool could be used as a single stage in a larger phylogenetic analysis pipeline, complementary to other methods. For example, the bootstrap values we obtain could be used as a fast and efficient way to obtain priors for a deeper Bayesian analysis, in order to gain further support for a particular topology or for parameter estimation.

One of the biggest challenges of this work was calculating invariants. We used methods in elimination theory to find a Gröbner basis with the software Macaulay2, but this does not scale well. Indeed, we were only able to calculate degree 2 invariants for the K3P model. This model is more versatile than K2P and JC, so we hope our results provide motivation for developing better methods of calculating invariants in this case. In Cummings et al. (2024), the authors reduce the calculations for finding quadratic invariants for 4-leaf 4-cycle networks under the Cavender-Farris-Neyman (CFN) 2-state model to finding the kernel of a linear map. The result is a much faster method of calculating invariants than using Gröbner basis methods and has been extended to higher degree invariants and other group-based models in Cummings and Hollering (2025), where the authors were able to calculate all minimal generators of the 4-leaf 4-cycle network under the K3P model up to degree 3. However, even the K3P model is somewhat simplistic, so we would like to be able to calculate invariants for more complex substitution models such as the generalized time reversible (GTR) model, or models that incorporate a molecular clock. Work in this direction has been recently performed for the CFN model on phylogenetic trees in Coons and Sullivant (2021), and recent theoretical results for the GTR model on phylogenetic trees (Casanellas et al. 2024) suggest that it may be possible to compute phylogenetic invariants for those models using similar methods to those we used here.

We currently do not have an interpretation of the invariants we have found in terms of the network topology. In the Supplementary Materials, we determine which of the invariants belong only to a single topology, and which are shared between different topologies, but we do not know what (if anything) they are telling us of the topology. Having a greater understanding of the invariants, or determining invariants that correspond to different topological features may enable faster convergence than we have observed here, or they may enable a multistep approach to inferring networks, in which first invariants are applied to find for example, the correct circular order, and once this is determined, different invariants could be applied to determine the reticulation vertex. Such a multistage approach would also enable the use of invariants of different degree. This is the topic of future work.

## Supplementary Material

syaf071_Supplemental_File

## Data Availability

All scripts used in this project, including scripts for simulating data and Macaulay2 scripts for calculating invariants, are available at the GitHub repository https://github.com/SR-Martin/4cycle_invariants. All simulated data and results presented are available at https://doi.org/10.5061/dryad.44j0zpcrk.
